# cChIP-seq: a robust small-scale method for investigation of histone modifications

**DOI:** 10.1186/s12864-015-2285-7

**Published:** 2015-12-21

**Authors:** Cristina Valensisi, Jo Ling Liao, Colin Andrus, Stephanie L. Battle, R. David Hawkins

**Affiliations:** Division of Medical Genetics, Department of Medicine, Department of Genome Sciences, Institute for Stem Cell and Regenerative Medicine, University of Washington School of Medicine, Seattle, WA USA; Turku Centre for Biotechnology, Turku, Finland

**Keywords:** ChIP-seq, Histone modifications, Epigenomic, Chromatin immunoprecipitation

## Abstract

**Background:**

ChIP-seq is highly utilized for mapping histone modifications that are informative about gene regulation and genome annotations. For example, applying ChIP-seq to histone modifications such as H3K4me1 has facilitated generating epigenomic maps of putative enhancers. This powerful technology, however, is limited in its application by the large number of cells required. ChIP-seq involves extensive manipulation of sample material and multiple reactions with limited quality control at each step, therefore, scaling down the number of cells required has proven challenging. Recently, several methods have been proposed to overcome this limit but most of these methods require extensive optimization to tailor the protocol to the specific antibody used or number of cells being profiled.

**Results:**

Here we describe a robust, yet facile method, which we named carrier ChIP-seq (cChIP-seq), for use on limited cell amounts. cChIP-seq employs a DNA-free histone carrier in order to maintain the working ChIP reaction scale, removing the need to tailor reactions to specific amounts of cells or histone modifications to be assayed. We have applied our method to three different histone modifications, H3K4me3, H3K4me1 and H3K27me3 in the K562 cell line, and H3K4me1 in H1 hESCs. We successfully obtained epigenomic maps for these histone modifications starting with as few as 10,000 cells. We compared cChIP-seq data to data generated as part of the ENCODE project. ENCODE data are the reference standard in the field and have been generated starting from tens of million of cells. Our results show that cChIP-seq successfully recapitulates bulk data. Furthermore, we showed that the differences observed between small-scale ChIP-seq data and ENCODE data are largely to be due to lab-to-lab variability rather than operating on a reduced scale.

**Conclusions:**

Data generated using cChIP-seq are equivalent to reference epigenomic maps from three orders of magnitude more cells. Our method offers a robust and straightforward approach to scale down ChIP-seq to as low as 10,000 cells. The underlying principle of our strategy makes it suitable for being applied to a vast range of chromatin modifications without requiring expensive optimization. Furthermore, our strategy of a DNA-free carrier can be adapted to most ChIP-seq protocols.

**Electronic supplementary material:**

The online version of this article (doi:10.1186/s12864-015-2285-7) contains supplementary material, which is available to authorized users.

## Background

Chromatin immunoprecipitation (ChIP) is the reference method for investigating protein-DNA interactions and chromatin-binding protein modifications, such as histone tail modifications. Genome-wide applications first coupled ChIP with microarrays (ChIP–chip) [1]. Then with the develop of next generation sequencing (NGS) technology, ChIP coupled with high-throughput sequencing (ChIP-seq) became the golden standard [2, 3]. While ChIP-seq offers several important improvements over the array-based application [4, 5], such as higher resolution, less noise and greater coverage, ChIP-seq still suffers from the limitation imposed by the large amount of cellular material needed for the chromatin immunoprecipitation step and amplification of the isolated DNA. Therefore, in the last few years a great deal of effort has been directed at developing ChIP-seq protocols to lower the scale by orders of magnitude.

ChIP-seq is a complex and multi-step process. The numerous steps with few quality control steps throughout contribute to the challenges when scaling ChIP-seq. In addition, limited amounts of chromatin are further challenged by non-specific interactions with beads and antibody. The signal-to-noise ratio, therefore, tends to decrease as the number of cells used for ChIP decreases. Previously, two methods were developed in an attempt to solve the issue of constructing libraries from small amounts of DNA obtained from ChIP on as few as 10,000 cells. Nano-ChIP-seq achieved success for several histone modifications using 10,000 cells by implementing a modified primer to first amplify the DNA by primer extension using Sequenase, followed by PCR amplification, then restriction digest to remove the primer/adaptor prior to standard library amplification [6]. This study also pointed out the need of titrating the quantities of antibody and beads for each mark, as optimizing antibody to beads and antibody-coated beads to chromatin are not linear in reduction [7–9], making these steps perhaps the greatest hurdle for small-scale ChIP. A single tube linear amplification method (LinDA) was recently developed and successful for ChIP-seq for H3K4me3 on 10,000 cells [10]. This method requires additional modifications prior to standard library preparation. T7 linkers are added for in vitro transcription and cDNA synthesis, which are subsequently removed by restriction digest prior to standard library preparation. Each of these methods, however, have yet to be widely adopted, perhaps due to the complex nature of the amplification schemes as well as the aforementioned need to optimize ChIP reaction conditions. This amplification complexity is potentially overcome by the use of whole genome amplification (WGA) approaches. This was illustrated for ChIP-seq of H3K4me2 largely optimized on chromatin equivalents of 10,000 cells, with Spearman’s correlation values for replicates ranging from 0.58 to 0.65. Slightly lower correlations were found for chromatin equivalents of 1000 cells [11].

The most complex, but scalable ChIP approach to date is iChIP (indexing-first chromatin IP) [12]. This method overcomes the issue of limited cells by pooling multiple cell populations together after on-bead indexing of the sonicated, chromatinized DNA. A sequential ChIP process is used with this intermediate indexing step. First, histone H3 is immunoprecipitated, as this is an abundant and genome-wide histone that provides a working scale ChIP reaction, which is followed by ligating barcode sequences that are later used to identify each cell population. Once multiple cell types are barcoded, they are pooled and subjected to paralleled histone modification ChIP-seq. Although this method allowed for ChIP on as few as 500 cells per population, the need for contextually assaying multiplexed cell populations limits the usage to large comparative studies.

The pooling of cell types to create a working scale ChIP reaction was originally described by O’Neill and colleagues by using *Drosophila* chromatin as a carrier [13], and therefore called carrier ChIP (cChIP), in order to ChIP limited numbers of mouse cells (10,000 - 100 cells). This has the advantage of establishing a single scale for ChIP because the bulk of input chromatin applies to the carrier. This is also advantageous when using multiple antibodies, as most function similarly at such a scale, and therefore optimization for each antibody is not needed. The overwhelming disadvantage of this method, as applied to ChIP-seq, is the presence of carrier DNA, which is not problematic when using species-specific primers for quantitative PCR, but will overwhelm sequencing libraries. Thus, making this approach unsuitable for ChIP-seq, but provides a basis for a working scale ChIP reaction for limited cell amounts. For example, a similar approach was taken for developing a small-scale ChIP-seq protocol using a bacterial DNA as a carrier to aid library preparation [14]. The caveat is that in order to get the sequencing depth necessary for profiling either histone marks or transcription factors the library needs to be sequenced to a substantially greater depth as up to 80 % of the reads mapped to the bacterial genome.

**Fig. 1:**
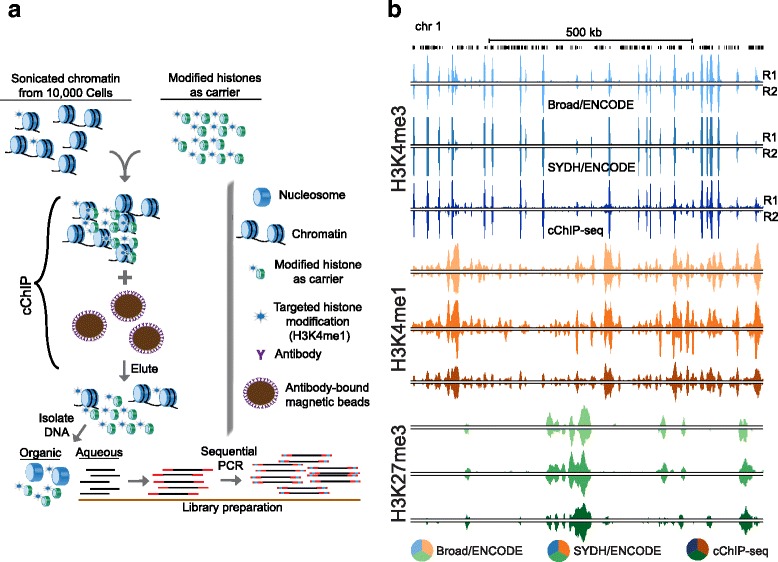
cChIP-seq. **a** The schematic illustrates conceptual design and key steps of the cChIP-seq protocol. **b** UCSC genome browser snapshot showing a representative example of cChIP-seq and ENCODE data for H3K4me3 (*shades of blue*), H3K4me1 (*shades of orange*) and H3K27me3 (*shades of green*) generated in the K562 cell line (chr17). R1: replicate 1; R2: replicate 2

Collectively, these approaches point out two disadvantages of low scale ChIP-seq, namely chromatin to beads to antibody ratio optimization and amplification of isolated DNA. The need to optimize the amount of antibody-coated beads is due to the fact that a disproportion between antibody and epitopes contributes to non-specificity, and therefore noise. cChIP [13], as well as iChIP-seq [12], overcome this by using a working scale ChIP reaction in the range of a few thousand to hundreds of cells. Our goal was to develop a method for ChIP-seq that does not require i) highly tailored optimization of chromatin to beads to antibody ratios and ii) extensive processing for the amplification of chromatin immunoprecipitated DNA. We developed cChIP-seq: carrier ChIP-seq (Fig. 1a and Methods). As illustrated in Fig. 1, this method is based on a widely utilized standard ChIP protocol [5], where the main modification is the introduction of a chemically modified recombinant histone H3 as the carrier. We reasoned that recombinant histones with a single chemical modification, matching that which is to be assayed, could serve as a “chromatin carrier” for the purpose of maintaining the working scale of the ChIP reactions. This removes the need to optimize the chromatin to antibody to beads ratios as a suitable number of modified histones are present as an epitope for the antibody. Furthermore, a DNA-free carrier does not require dealing with unwanted DNA during library preparation and sequencing. We show that cChIP-seq is highly successful at generating data on 10,000 cells for several key histone modifications, and requires little modification to a standard ChIP-seq protocol.

## Results

To establish cChIP-seq (Fig. 1a), we optimized sonication of a limited number of crosslinked cells down to 30,000 cells using the Covaris LE220 ultrasonicator (Additional file 1: Figure S1). For each test, cells were counted prior to chromatin isolation, as were nuclei prior to sonication to ensure cell and chromatin amounts in each cChIP experiment. We estimated the amount of recombinant carrier histone based on potentially marked histones (see Methods). We have applied cChIP-seq to three informative and commonly investigated histone modifications: H3K4me3, H3K4me1 and H3K27me3 (Fig. 1b). We have compared our data to existing data from either the ENCODE consortium or Roadmap Epigenomics Consortium (REC). We provide a robust, yet simple method for ChIP-seq of 10,000 cells, which should be applicable to almost any histone modification and compatible with most working ChIP protocols.

As an initial optimization, we performed cChIP-seq for H3K4me3 in K562 cells (Fig. 1b), reasoning that it is the most robust mark to ChIP and should perform best at a small scale. After chromatin sonication, we mixed 10,000, 5000, 500 and 100 whole-cell equivalents with recombinant histone H3 with a lysine 4 trimethylation modification (recH3K4me3) and incubated with magnetic beads pre-bound with antibody against H3K4me3. We then proceeded to perform ChIP-seq as described previously [8, 9], but with the minor modification of generating libraries using PCR amplification performed in two sequential rounds of limited cycles to help reduce amplification-based background (see Methods). We generated a total of ~ 150 million monoclonal mapped reads so that all libraries were likely sequenced to saturation. We assessed correlations between replicates and across cell amounts, followed by a comparison to ENCODE consortium data as a standard for the field [15]. In order to account for differences that arise from variation in lab-to-lab practices, we compared our data to two different replicated datasets from ENCODE: Broad and SYDH. In order to avoid bias due to differences in the computational analysis, we obtained raw data for each datasets (two replicates for each assay to match our data) and analyzed all datasets with our pipeline under the same settings (see Methods).

**Fig. 2:**
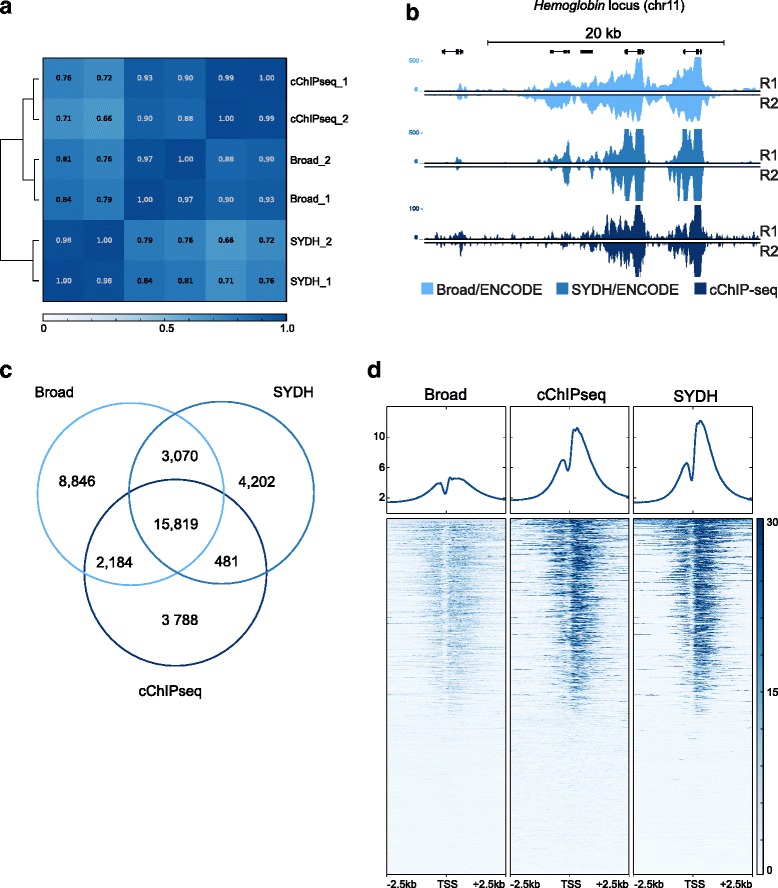
cChIP-seq for H3K4me3 on 10,000 cells in K562 cells. **a** Pearson’s correlation values heatmap for replicates of cChIP-seq on 10,000 cells and ENCODE data. **b** UCSC genome browser snapshot showing H3K4me3 signal (RPKM input normalized) for the three datasets at the hemoglobin locus (chr11). **c** Venn diagram showing the number of unique and common regions enriched for H3K4me3 across cChIP-seq, Broad and SYDH datasets. **d** Heatmap representation of the signal intensity (RPKM input normalized) across the three datasets in a 5 kb window centered at all protein-coding transcription start sites (TSS) [GENCODE assembly GRCh37]. R1: replicate 1; R2: replicate 2

cChIP-seq replicates for 10,000 cells correlated as well as replicates for each ENCODE group (Fig. 2a and b). Pearson’s correlation coefficients calculated across cChIP-seq, Broad and SYDH showed that at 10,000 cells cChIP-seq performed well relative to ENCODE data with average coefficients of 0.90 with respect to Broad and 0.7 with respect to SYDH. The average Pearson’s correlation coefficient between Broad and SYDH was ~0.80 (Fig. 2a, Additional file 1: Figure S2a). For lower cell numbers we did not obtain data of sufficient quality (Additional file 1: Figure S2a and b). Although enrichment for H3K4me3 at promoter regions could be observed when using 5000 cells (Additional file 1: Figure S2a), the Pearson’s correlation coefficient with respect to ENCODE data was less than 0.60 (Additional file 1: Figure S3b). This led us to conclude that below 10,000 cells cChIP-seq was sub-optimal. Given our goal to develop a protocol with minimal optimizations per mark or per starting amount of cells, we focused on 10,000 cells for the remaining validation of cChIP-seq. In addition, visual inspection across the genome of H3K4me3 cChIP-seq data using 10,000 cells, demonstrates that our data is highly similar to ENCODE data (Fig. 2b) further supporting the reliability of our method.

We compared cChIP-seq data to ENCODE data with respect to peak recovery. We used MACS to call peaks on each replicate of cChIP-seq and ENCODE data [16]. We called a similar number of peaks across the three datasets, and each replicate recovered a similar overlap for each group (Additional file 1: Figure S3c). Overlap of cChIP-seq peaks recovered approximately 80 % of the peaks called in either replicate. Similar peak overlaps were found for ENCODE replicates (Broad: 74 % average replicate overlap; and SYDH: 88 % average replicate overlap). Next, we compared the peaks called on merged replicates across the three datasets. When comparing cChIP-seq peaks to both Broad and SYDH, 71 % of the cChIP-seq peaks were found in both ENCODE datasets (Fig. 2c). Asking the reverse, cChIP-seq recovered 60 % of the Broad peaks and 67 % of the SYDH peaks, performing as well as the ENCODE datasets when compared to each other (capturing 63 and 80 % of the other’s peaks; Fig. 2c). Only 17 % of cChIP-seq peaks were unique to cChIP-seq, similar to SYDH (17.8 %) and lower than Broad (29.6 % unique). We also observed a similar enrichment of the signal across datasets at Gencode transcription start sites (TSS), regardless of peak calls (Fig. 2d). To confirm this observation we determined the overlap of peaks across the three datasets at TSS. We observed that over 90 % of the TSSs captured by each datasets were also enriched for H3K4me3 in the other two datasets. These results indicate that cChIP-seq is a robust method to profile H3K4me3 marks using three orders of magnitude fewer cells with respect to what was used for generating ENCODE data. Based on the three-way comparison across groups, we conclude that differences between cChIP-seq and ENCODE data are likely to due more to expected lab-to-lab variability rather than operating at a lower scale (Fig. 2c). Furthermore, the use of modified carrier histones results in a simple method that does not require any upfront optimization to scale down ChIP reaction conditions.

**Fig. 3:**
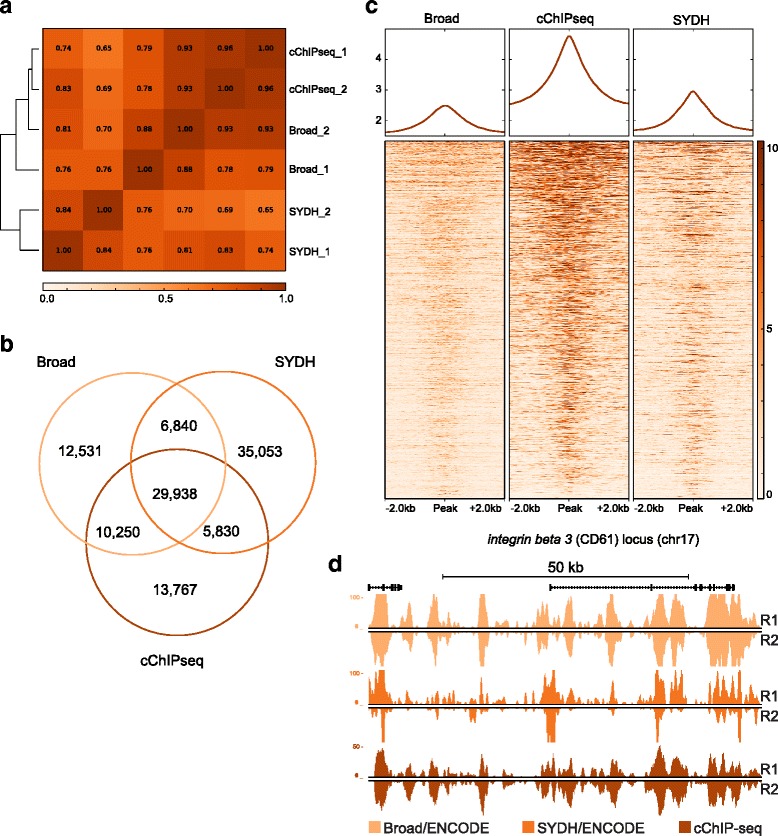
cChIP-seq for H3K4me1 on 10,000 cells in K562 cells. **a** Pearson’s correlation values heatmap for replicates of cChIP-seq on 10,000 cells and ENCODE data. **b** Venn diagram showing the number of unique and common regions enriched for H3K4me1 across cChIP-seq, Broad and SYDH datasets. **c** Heatmap representation of the signal intensity (RPKM input normalized) across the three datasets in a 4 kb window centered at all H3K4me1 enriched regions called in the three datasets (the entirety of peaks shown in 3b. **d** UCSC genome browser snapshot showing H3K4me1 signal (RPKM input normalized) for the three datasets at the *integrin beta 3* (*CD61*) locus (chr17). R1: replicate 1; R2: replicate 2

We next sought to apply our method to other histone modifications as well as test the performance on a different cell line. We first applied cChIP-seq to profile H3K4me1, a highly cell-type specific mark [7] associated with enhancer regions [17]. H3K4me1 enrichment in K562 was highly comparable to ENCODE data (Fig. 1b). Pearson’s correlation across cChIP-seq, Broad and SYDH confirmed that our method was highly reproducible (*r* = 0.96) and correlated with both ENCODE datasets (*r* = 0.86 with respect to Broad; *r* = 0.76 with respect to SYDH) as well as ENCODE datasets correlated with each other (*r* = 0.73) (Fig. 3a, Additional file 1: Figure S2b). Next, we compared peaks called in the two replicates per each datasets and observed that correlation across replicates was again comparable to ENCODE replicates (Fig. 3a, Additional file 1: Figure S4a). Moreover, cChIP-seq identified the same numbers of putative enhancers (over 40,000 when considering peaks shared between replicates) as predicted by either ENCODE dataset in this cell line (Additional file 1: Figure S4a). After replicates were merged, each datasets shared an average of 47 % of the peaks (29,938) with both the other two datasets (Fig. 3b). This drop in percent overlap compared to H3K4me3 may be due to challenges calling broader H3K4me1 peaks. When asking how many of the ENCODE peaks cChIP-seq identified, we observed that our method recovered 81 % of the peaks called in both Broad and SYDH (out of 36,778). Again we observed that the cChIP-seq data performed well with respect to either ENCODE dataset, and as well as ENCODE datasets performed with respect to each other confirming that differences between cChIP-seq and ENCODE data are due to inter-laboratory variability rather than the operating scale.

Next, we compared the signal intensity of the three datasets in a 4 kb window centered at all H3K4me1 enriched regions called in the three datasets (Fig. 3c). The three datasets performed similarly at all peaks regardless of peak calls. We did observe a higher baseline signal across our data with respect to ENCODE and a similar trend was observed for this mark in H1 hESC (see below. Additional file 1: Figure S5e and f); however, the signal-to-noise ratio is more than adequate for accurately calling peaks. To further confirm our results on H3K4me1, we compared the signal intensity of cChIP-seq and ENCODE data at K562 enhancers previously predicted by RFECS, a Random-Forest based algorithm recently developed to identify enhancers based on several histone modifications and p300 localization [18]. All datasets showed some degree of enrichment at RFECS enhancer locations (Additional file 1: Figure S4b) as well as a similar fraction of RFECS enhancers captured (on average, 57 %) (Additional file 1: Figure S4c). Overall, data generated for H3K4me1 on three orders of magnitude fewer cells by cChIP-seq performed in a highly comparable manner with respect to ENCODE data (Fig. 3d).

To ensure that cChIP-seq has applicability for various cell types, we performed cChIP-seq on 10,000 cells for H3K4me1 in H1 hESCs and compared these data to those previously generated by the REC on a few million cells [8, 9]. Both visual inspection of the global enrichment and the Pearson’s correlation between replicates (*r* = 0.96), indicate that cChIP-seq replicates are highly reproducible (Additional file 1: Figure S5a and b). Furthermore, cChIP-seq data correlate well with REC data (*r* = 0.76) (Additional file 1: Figure S5b). We observed that 63 % of the REC H3K4me1 peak calls on merged replicates were recovered by cChIP-seq (Additional file 1: Figure S5c). However, more than half of peaks called on cChIP-seq data appeared to be unique. We reasoned that at least a fraction of those unique peaks could have been captured by either of the REC ChIP-seq replicates. When we specifically looked for overlap between the unique cChIP-seq and either of the REC replicates (Additional file 1: Figure S5d), we found than 11 % of the unique cChIP-seq peaks (4878) overlapped peaks called in REC replicate 1 that were not called on the merged replicates. Similarly, an additional 19 % of the unique cChIP-seq (8430) were captured by REC replicate 2 that were not called on the merged replicates. Considering the total number of REC peaks captured by cChIP-seq, we concluded that our data correlated well with data previously generated on few million cells. This is supported by comparing the signal intensity of both datasets in a 4 kb window centered at H3K4me1 enriched regions for REC ChIP-seq (Additional file 1: Figure S5e). We also compared enrichment in both datasets on RFECS-predicted enhancers in H1 cells [18]. While both data showed enrichment at these sites, we observed higher signal in cChIP-seq compared to data ChIP-seq both at and around the peaks (Additional file 1: Figure S5f). Finally, we sought to measure how many RFECS-predicted enhancers [18] were captured by cChIP-seq as compared to REC data. We observed that cChIP-seq H3K4me1 peaks overlapped 61 % (33,996 out of 55,382) of RFECS enhancers, while REC data captured 45 % (24,831) of RFECS enhancers. Furthermore, cChIP-seq captured all the RFECS enhancers captured by ChIP-seq. Altogether, we have shown that cChIP-seq method successfully scaled our previous ChIP-seq protocol [8, 9] by two orders of magnitude for H3K4me1 in H1 hESC line.

**Fig. 4:**
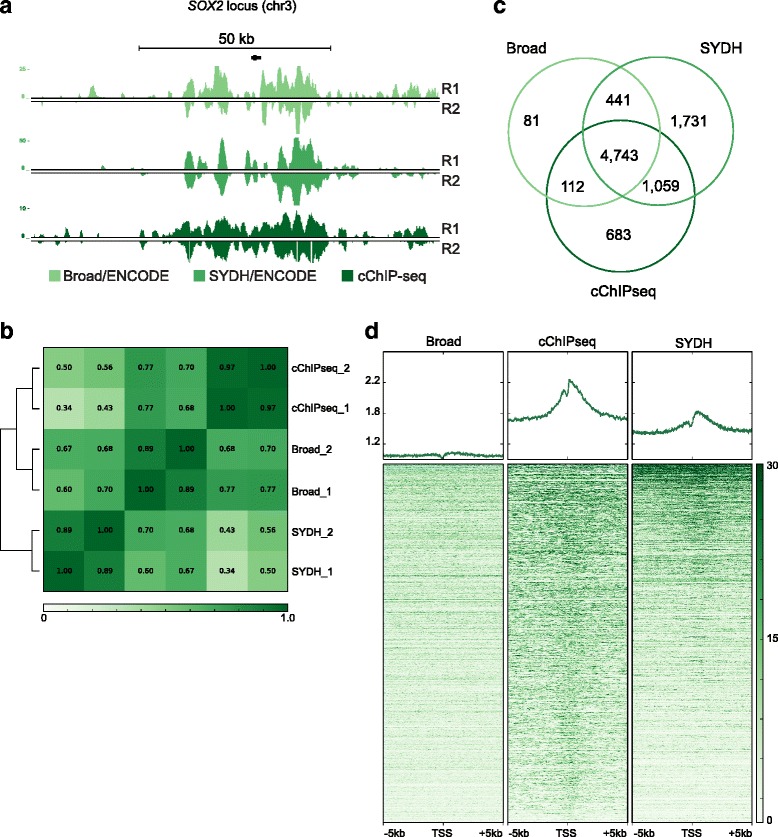
cChIP-seq for H3K27me3 on 10,000 cells in K562 cells. **a** UCSC genome browser snapshot showing H3K27me3 signal (RPKM input normalized) for cChIP-seq and ENCODE data at the *SOX2* locus (chr3). R1: replicate 1; R2: replicate 2. **b** Pearson’s correlation values heatmap for replicates of cChIP-seq on 10,000 cells and ENCODE data. **c** Venn diagram shows the number of unique and common regions enriched for H3K27me3 across cChIP-seq, Broad and SYDH datasets. **d** Heatmap representation of the signal intensity (RPKM input normalized) across cChIP-seq and ENCODE data in a 10 kb window centered at protein-coding transcription start sites (TSS) [GENCODE assembly GRCh37] overlapping ChromaBlocks domains found in any of the three datasets (10,736 TSSs are shown)

Finally we tested cChIP-seq for the Polycomb repressive complexes-associated modification H3K27me3 in the K562 cell line. We generated H3K27me3 data on 10,000 cells using cChIP-seq and compared our data to both Broad and SYDH ENCODE datasets (Fig. 1b and Fig. 4a). Pearson’s correlation coefficients calculated across the three datasets indicated (Fig. 4b, Additional file 1: Figure S2c) that our replicates were highly reproducible (*r* = 0.97). While the correlation with the Broad dataset was good (*r* = 0.73), our data correlated less well with SYDH dataset (*r* = 0.46). The Pearson’s correlation coefficient between the two ENCODE datasets was 0.66 (Fig. 4b). We used ChromaBlocks, an algorithm previously developed to determine broad domains of histone modifications such as H3K27me3 [8], and called domains on merged replicated. When we merged all the domains called in cChIP-seq and ENCODE data (Fig. 4c), we identified 4743 broad H3K27me3 enriched regions common to all three datasets, accounting for 72 % of all enriched regions in cChIP-seq data. Similarly, 88 % of Broad H3K27me3 enriched regions and 59 % of SYDH regions were shared with both the other two datasets. We next looked at the global distribution of the signal in a 10 kb window around those protein-coding TSS that we found enriched for H3K27me3 in all the three datasets (Fig. 4d). Although the cChIP-seq and Broad data showed a better global correlation (Fig. 4b), the cChIP-seq and SYDH data showed a greater degree of enrichment around TSSs. Overall, these results indicate that cChIP-seq successfully generated H3K27me3ChIP-seq data using 10,000 cells.

## Discussion

In the last few years, there has been an upsurge in the use of ChIP-seq to map histone modifications in various cells. If we are to continue at this rate and to explore new cell types, we will need to profile rare cell populations whose abundance is limited. To aid in that endeavor we developed a robust, yet facile method for performing ChIP-seq on 10,000 cells, which includes sonication of limited cell amounts and does not require any advanced amplification scheme. We applied this to three of the most informative histone modifications as a measure of applicability to a broad set of modifications. Data generated with cChIP-seq were highly comparable to ENCODE and REC data. In comparison with other methods of scale [6, 10–12] cChIP-seq bypasses the challenges of optimizing ChIP-seq when scaling the number of cells by utilizing a recombinant histone carrier with a single modification corresponding to the mark to be assayed in ChIP-seq without the need to alter any of the upstream or downstream steps of the ChIP-seq procedure. Given that our strategy is based on matching the recombinant histone carrier with the histone modification to be assayed, we anticipate few hurdles in applying cChIP-seq to other types of histone modifications, such as acetylation, to further expand the number of chromatin modifications that can be surveyed using cChIP-seq. Furthermore, a similar approach was adopted for ChIP-seq targeting transcription factors [19], confirming the importance of a chromatin-like carrier. The nucleic acid-free recombinant carrier reported here for cChIP-seq provides advantages over other methods in that there is no need for removal of the carrier [14, 19], either in vitro or in silico. Therefore, this approach can be easily and readily adopted into any ChIP-seq protocol without requiring further modification or optimization.

Chromatin modifications are key for distinguishing regulatory elements [7, 17]. The use of histone modifications to identify previously unknown regulatory elements, such as promoters and enhancers, has also aided our understanding of disease associated genetic variant in the genome [20, 21]. Combinations of modifications are also informative about active versus poised states of these elements [22–25]. The recent advances in various genomic applications, such as ATAC-seq for open chromatin accessibility [26, 27], provide the unprecedented opportunity for profiling epigenomes moving away from cell lines and towards small cell populations in complex tissues. In line with this trend cChIP-seq offers a robust approach to access various histone modification landscapes in limited cell populations.

## Conclusions

In summary, cChIP-seq proved to generate highly reproducible, quality ChIP-seq data for multiple histone modifications, and should be applicable to a broad array of histone modifications. Coupling cChIP-seq with more complex library amplification schemes may prove useful for lower cell amounts, but would likely require substantial optimization of the amplification. In conclusion, the simplicity of cChIP-seq should make this method easily and widely adopted when applying to 10,000 cells.

## Methods

### Optimization of sonication conditions

K562 cells were counted before crosslinking. 250 × 10^3^, 125 × 10^3^, 62 × 10^3^, and 31 × 10^3^ cells were crosslinked in 1 % formaldehyde for 10 min at room temperature. After quenching with glycine and washing in cold PBS, cells were lysed in 1 ml of lysis buffer (50 mM HEPES KOH pH 7.5, 140 mM NaCl, 1 mM EDTA, 10 % glycerol, 0.5 % Nonidet P-40, and 0.25 % Triton X-100 supplemented with protease inhibitors). Nuclei were washed in 1 ml of washing buffer (10 mM Tris pH 8, 200 mM NaCl, 1 mM EDTA pH 8, 0.5 mM EGTA, supplemented with protease inhibitors) followed by a rinse in TE buffer supplemented with protease inhibitor. Nuclei were counted to verify the amount per each sample and resuspended in 135 μl of TE buffer supplemented with 0.1 % Triton X-100 and protease inhibitors. Nuclei were sonicated using Covaris LE220 ultrasonicator for 20, 25 and 30 min. Debris were removed and an aliquot of chromatin for each samples underwent reverse crosslinking at 65 °C O/N. Afterwards, DNA was purified using 24:1 chloroform/isoamyl alcohol (PCI). DNA was then quantified using Qubit HS dsDNA assay (Life Technologies) and fragmentation size distribution was tested using Agilent High Sensitivity DNA Assay.

### cChIP-seq

To determine the amount of carrier histone, we estimated the number of nucleosomes covered by ChIP-seq peaks, with each nucleosome plus linker occupying 200 bp under the peak. This estimate was used to calculate the number of modified histone H3 molecules from three million cells – our standard ChIP scale. We made an initial approximation for H3K4me1, the most abundant of modifications tested in cChIP-seq, and applied it to all modifications. From previously generated data we estimated ~400,000 modified nucleosomes under H3K4me1 peaks. We assume each histone per nucleosome is a) modified and b) a target for the antibody. This results in 800,000 histone targets for antibodies. To determine the amount of carrier that represents 800 k histones and three million cells per ChIP as our standard working scale we calculated the following:

$$ \begin{array}{c}\frac{\left(\#\ \mathrm{of}\ \mathrm{modified}\ \mathrm{histone}\mathrm{s}\right)\times \left(\#\ \mathrm{cells}\ \mathrm{ChIP}'\mathrm{d}\right)\times \left(\mathrm{molecule}\ \mathrm{weight}\ \mathrm{of}\ \mathrm{histone}\ \mathrm{H}3\right)}{\left(6.02213665168\mathrm{E}+23\mathrm{kDa}/\mathrm{kg}\right)} = \frac{\left(800,000\right)\times \left(3,000,000\right)\times \left(15.2\mathrm{kDa}\right)}{\left(6.02213665168\mathrm{E}+23\mathrm{kDa}/\mathrm{kg}\right)}\\ {} = 6.06\mathrm{e}-11\mathrm{kg}\ \mathrm{or}\ 60.6\mathrm{ng}\ \mathrm{of}\ \mathrm{carrier}\ \mathrm{histone}.\end{array} $$

After chromatin sonication, 10,000, 5000, 500 and 100 whole-cell equivalents were incubated with Dynabeads (Life Technologies) pre-bound with the specific antibody in 200 μl of binding buffer (10 mM Tris pH 8, 1 mM EDTA pH 8, 2 % Triton X-100, 0.2 % DOC, supplemented with protease inhibitors). For all cChIP-seq on 10,000 cells, experiments were performed in duplicate on independent batches of cells. Recombinant histone (Active Motif) with a single chemical modification matching that which is to be assayed was added and the mixture was incubated at 4 °C O/N. Based on our approximation of marked histones within nucleosomes covered by peaks, 60 ng of recombinant histone was used throughout all the experiments. Throughout all the experiments, the same amount of magnetic beads (11 μl) and antibody (3 μg) were used. The following antibodies were used: H3K4me3 (Active Motif), H3K4me1 (Diagenode) and H3K27me3 (Active Motif). Beads were washed 8 times with 200 μl of washing buffer (50 mM HEPES KOH pH 7.5, 1 mM EDTA, 1 % Nonidet P-40, 0.7 % DOC, 0.5 M LiCl, supplemented with protease inhibitors), followed by a wash with TE. Chromatin was eluted by incubating beads in TE supplemented with 1 % SDS at 65 °C for 20 min. Afterwards, chromatin underwent reverse crosslinking at 65 °C O/N and DNA was purified using 24:1 chloroform/isoamyl alcohol (PCI). All DNA recovered from an IP (“ChIPed” DNA) along with matched chromatin input were used for preparing Illumina-compatible libraries as previously described [8] with the following modifications. AMPure XP beads (Beckman Coulter) were used for reaction purification. Illumina-compatible adapters (custom design) were used at 7 nM final concentration. After ligation, two round of purification using AMPure XP beads were performed followed by 4 cycles of PCR using KAPA HiFi (Kapa Biosystems). After size-selection of 300 to 600 bp fragments on 2 % agarose gel, purified samples underwent to an average of 6 cycles of PCR, depending on the recovered amount of DNA after gel extraction. All libraries were sequenced in NextSeq 500 (Illumina) performing 1 × 75 cycles.

### ChIP-seq analysis

Raw sequence reads from ENCODE project were downloaded from http://hgdownload.cse.ucsc.edu/goldenPath/hg19/encodeDCC/wgEncodeBroadHistone/ and http://hgdownload.cse.ucsc.edu/goldenPath/hg19/encodeDCC/wgEncodeSydhHistone/, Broad and SYDH data respectively. All sequenced reads, both cChIP-seq and ENCODE data, were analyzed with the following pipeline and settings. Sequence reads were aligned to genome (version hg19) using Bowtie2 algorithm (settings: −N 1 -L 25). Uniquely mapping reads with quality score higher than 29 were retained. Pearson’s correlation heatmaps (bamCorrelate bins, −-corMethod pearson) and heatmap representations of signal intensity (computeMatrix reference-point followed by heatmapper in the multiheatmapper branch) were generated using deepTools suite [28]. Due to the large number of values per sample, the computation of our Pearson’s correlation coefficients p-values for these correlations rounded to 0 when calculated with R. To show the confidence of our Pearson’s correlation coefficients we calculated accompanying 95 % confidence intervals using Fisher’s r to z transformation to then find intervals for our coefficients over a normal distribution (Additional file 1: Figure S2). For the UCSC genome browser tracks, ChIP-seq signals were normalized by RPKM values of input and ChIP-seq sample followed by subtraction of input from ChIP using deepTools suite (bamCompare, −-normalizeUsingRPKM --ratio subtract --ignoreDuplicates). For H3K4me1 and H3K4me3, peaks were called using MACS v1.4 using the --nomodel mode [16]. For H3K27me3, domains (broad peaks) were called using ChromaBlocks [8] using the R package Repitools (http://bioconductor.org/packages/release/bioc/html/Repitools.html - settings: ipWidth = 100, inputWidth = 500 and preset =“large”) [29]. For identifying regions that were enriched for a specific modification across datasets or between replicates, peaks were overlapped using mergePeaks (Homer suite) [30]. Peaks overlapping for at least 1 bp are merged into shared enriched regions by mergePeaks. Venn diagrams throughout this work show the number of unique peaks and merged peaks as defined by mergePeaks. Pie chart in Additional file 1: Figure S4d was generated by counting (w/o merging) the number of H3K4me1 cChIP-seq peaks that overlapped at least one time with H3K4me1 REC peaks called either on merged replicates or on single replicates. Only peaks called on either single replicate that were not called after merging replicates were counted as peaks called on single replicates.

## Availability of supporting data

All data have been deposited to the Sequence Read Archive (SRA) under accession number SRS972820.

## Additional file

Additional file 1:
**The following additional data are available with the online version of this paper.** Additional data file 1 contains the following figures. **Figure S1.** illustrates the optimization on sonication down to 30,000 crosslinked cells using the Covaris LE220 ultrasonicator. **Figure S2.** provides p-values for the Pearson’s correlation heatmaps shown in Figs. 2, 3 and 4. **Figure S3.** relates to Fig. 2 and provides additional results for H3K4me3 in K562 cell line. **Figure S4.** relates to Fig. 3 and provides additional results for H3K4me1 in K562 cell line. **Figure S5.** shows results obtained for H3K4me1 in H1 hESC line. (PDF 3493 kb)
