# Supplementary material for: cChIP-seq: a robust small-scale method for investigation of histone modifications
Source: BMC Genomics. 2015 Dec 21;16:1083. doi: 10.1186/s12864-015-2285-7 (PMC4687106; doi:10.1186/s12864-015-2285-7)
Supplement: Additional file 1: — The following additional data are available with the online version of this paper. Additional data file 1 contains the following figures. Figure S1. illustrates the optimization on sonication down to 30,000 crosslinked cells using the Covaris LE220 ultrasonicator. Figure S2. provides p-values for the Pearson’s correlation heatmaps shown in Figs. 2, 3 and 4. Figure S3. relates to Fig. 2 and provides additional results for H3K4me3 in K562 cell line. Figure S4. relates to Fig. 3 and provides additional results for H3K4me1 in K562 cell line. Figure S5. shows results obtained for H3K4me1 in H1 hESC line. (PDF 3493 kb) [file 12864_2015_2285_MOESM1_ESM.pdf]

Figure S1

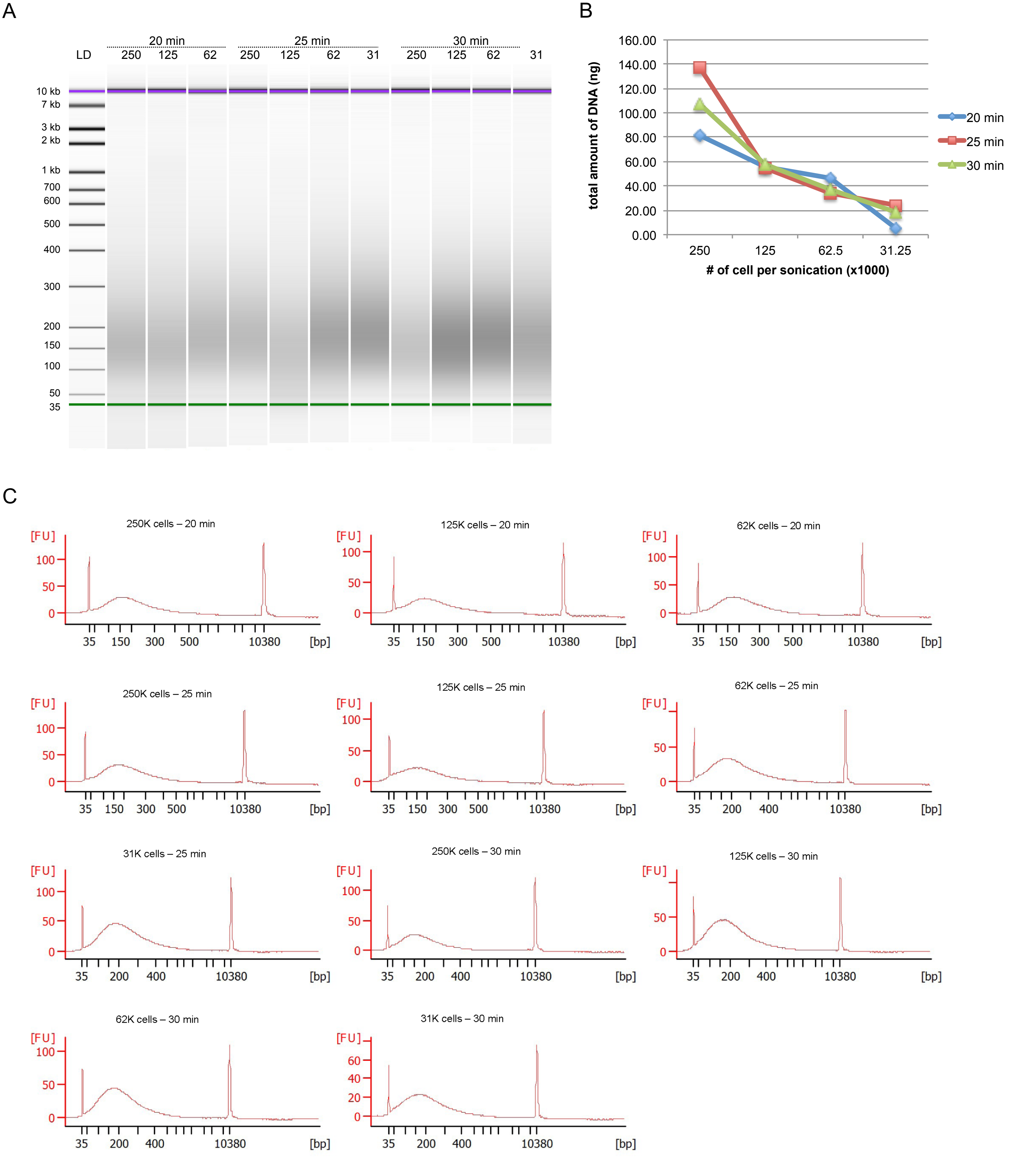

Figure S1. Sonication for low amounts of cells. A) Agilent High Sensitivity DNA Assay-generated gel-like picture showing DNA fragment length distribution for 31,000 to 250,000 crosslinked K562 cells sonicated using the Covaris LE220 ultrasonicator for 20, 25 and 30 minutes. B) After DNA purification, total amount of DNA recovered per each samples is shown. C) Detailed profiles of the samples shown in a.

Figure S2

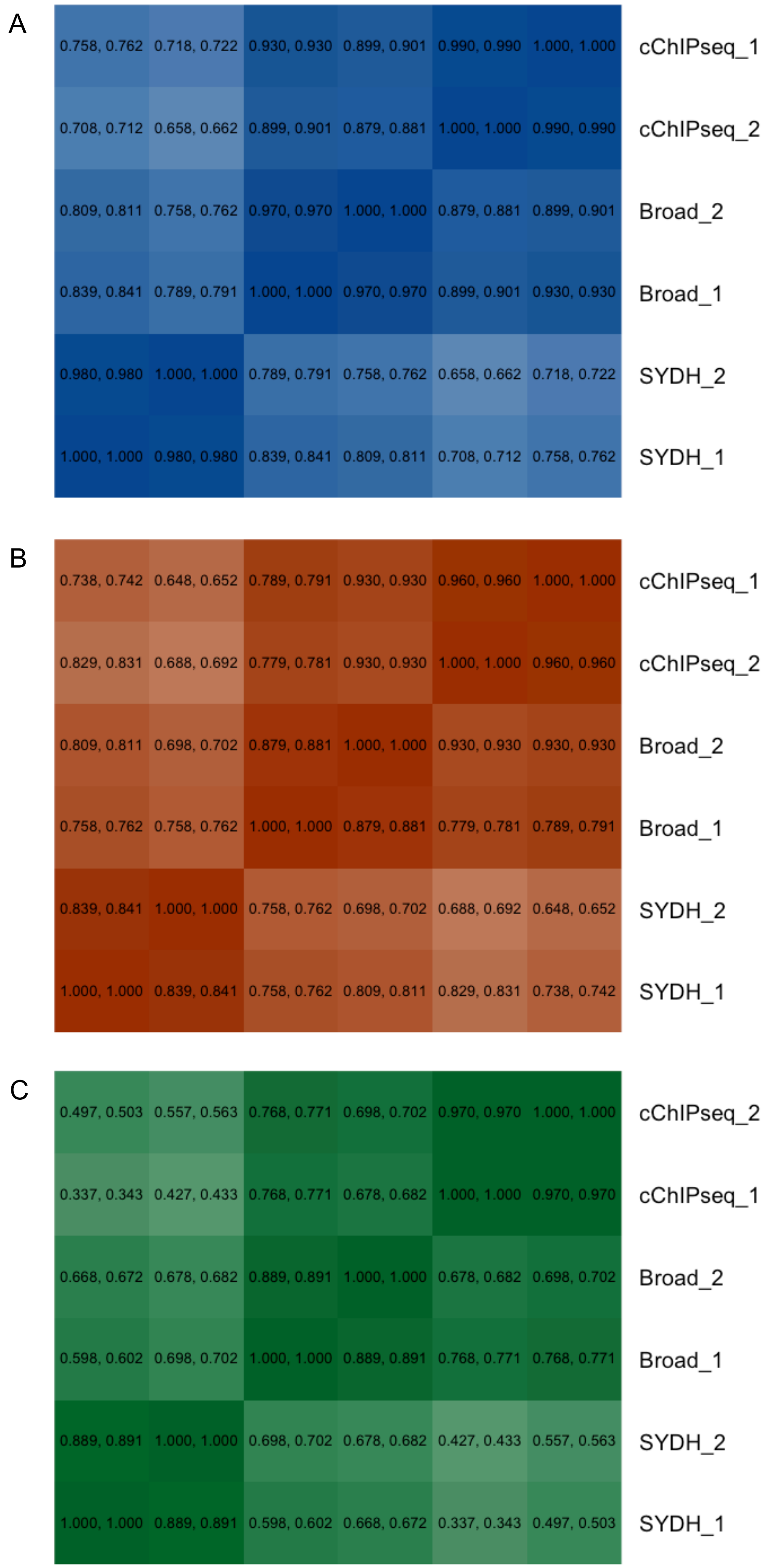

Figure S2. Confidence intervals for Pearson's correlation. Heatmap representation of the 95% confidence intervals (lower, upper) for each Pearson's correlation test among cChIP-seq, Broad and SYDH data**.** A) H3K4me3; refers to Figure 2a. B) H3K4me1; refers to Figure 3a. C) H3K27me3; refers to Figure 4b.

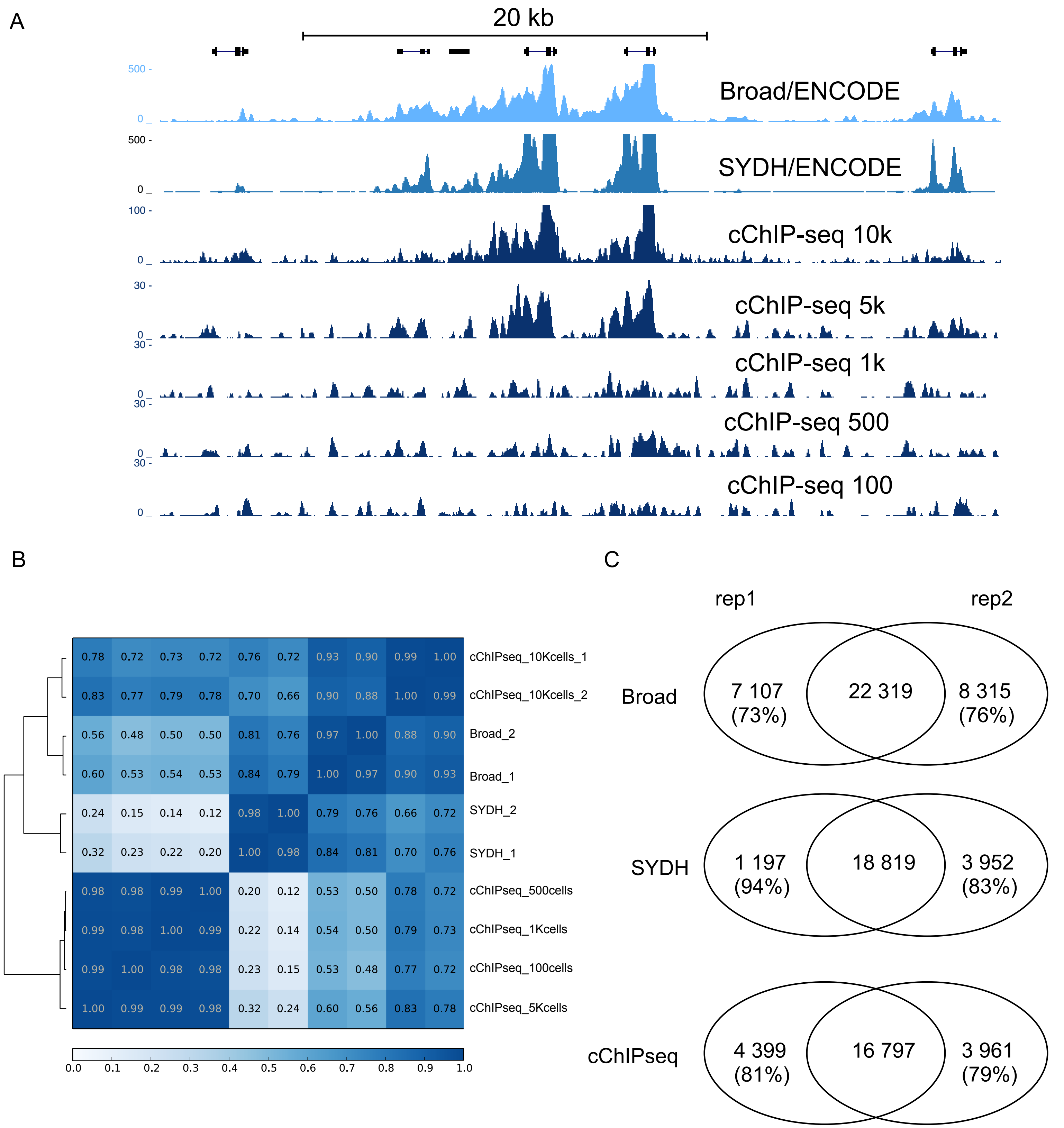

Figure S3. **cChIP-seq on 10,000; 5,000; 1,000; 500 and 100 cells for H3K4me3 in the K562 cell line. A**) UCSC genome browser snapshot showing H3K4me3 signal (RPKM input normalized) for cChIP-seq and ENCODE data, single replicates, at the hemoglobin locus (chr11). **B**) Pearson's correlation values heatmap for cChIP-seq, ENCODE Broad and SYDH data. **C**) Venn diagrams showing the number of unique and common regions enriched for H3K4me3 for Broad, SYDH and cChIP-seq replicates respectively. In parenthesis is the percentage of unique regions per each replicate.

a

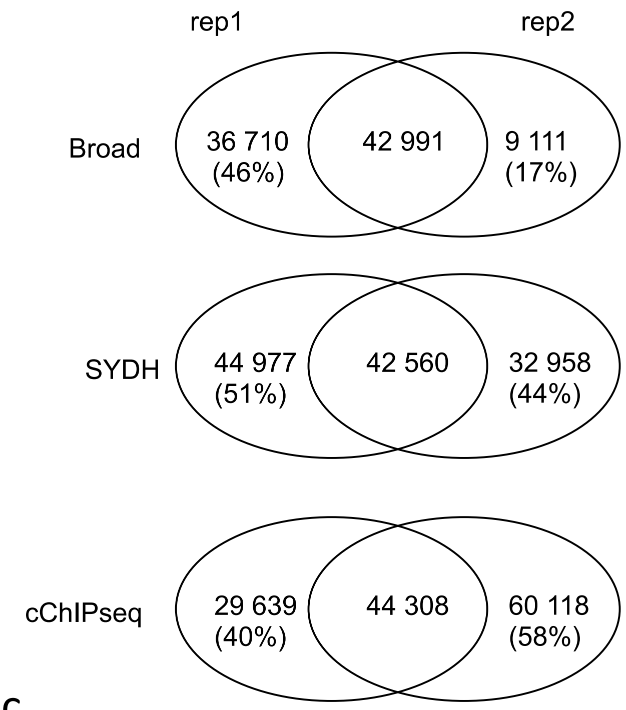

b

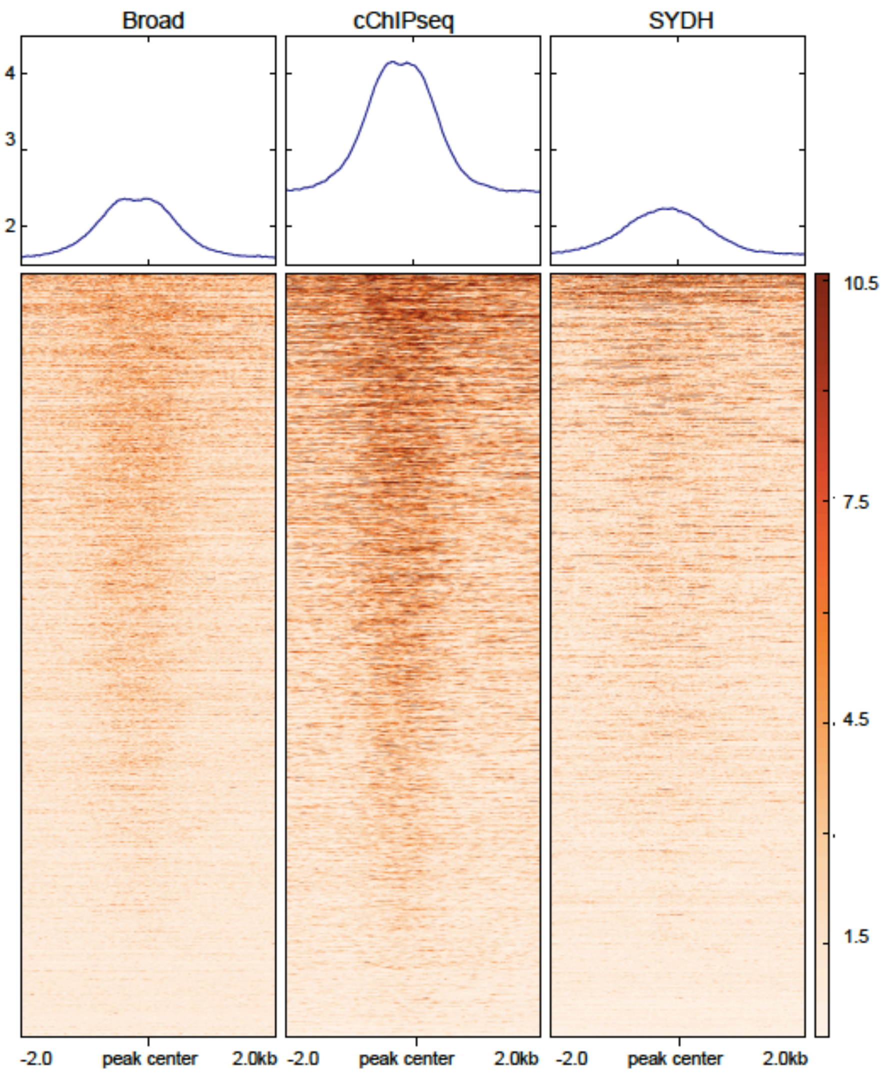

c

RFECS-predicted enhancers (81,936) captured by each datasets

	#	%	% shared
cChIP-seq	44,857	55%	74%
Broad	51,047	62%	65%
SYDH	43,687	53%	75%

Figure S4. cChIP-seq on 10,000 cells for H3K4me1 in the K562 cell line. A) Venn diagrams showing the number of unique and common regions enriched for H3K4me1 for Broad, SYDH and cChIP-seq replicates respectively. In parenthesis is the percentage of unique regions per each replicate. B) Heatmap representation of the signal intensity (RPKM input normalized) across the three datasets in a 4 kb window centered at K562 enhancers predicted by RFECS (81,936 regions). C) Table reports the number (#) as well as the percentage (%) respect to the total of RFECS-predicted enhancers in K562 (81,936 regions in total) that are captured by peaks called in each datasets. The third column (% shared) reports the percentage respect to the total of captured RFECS enhancers that are captured also by both the other two datasets.

Figure S5

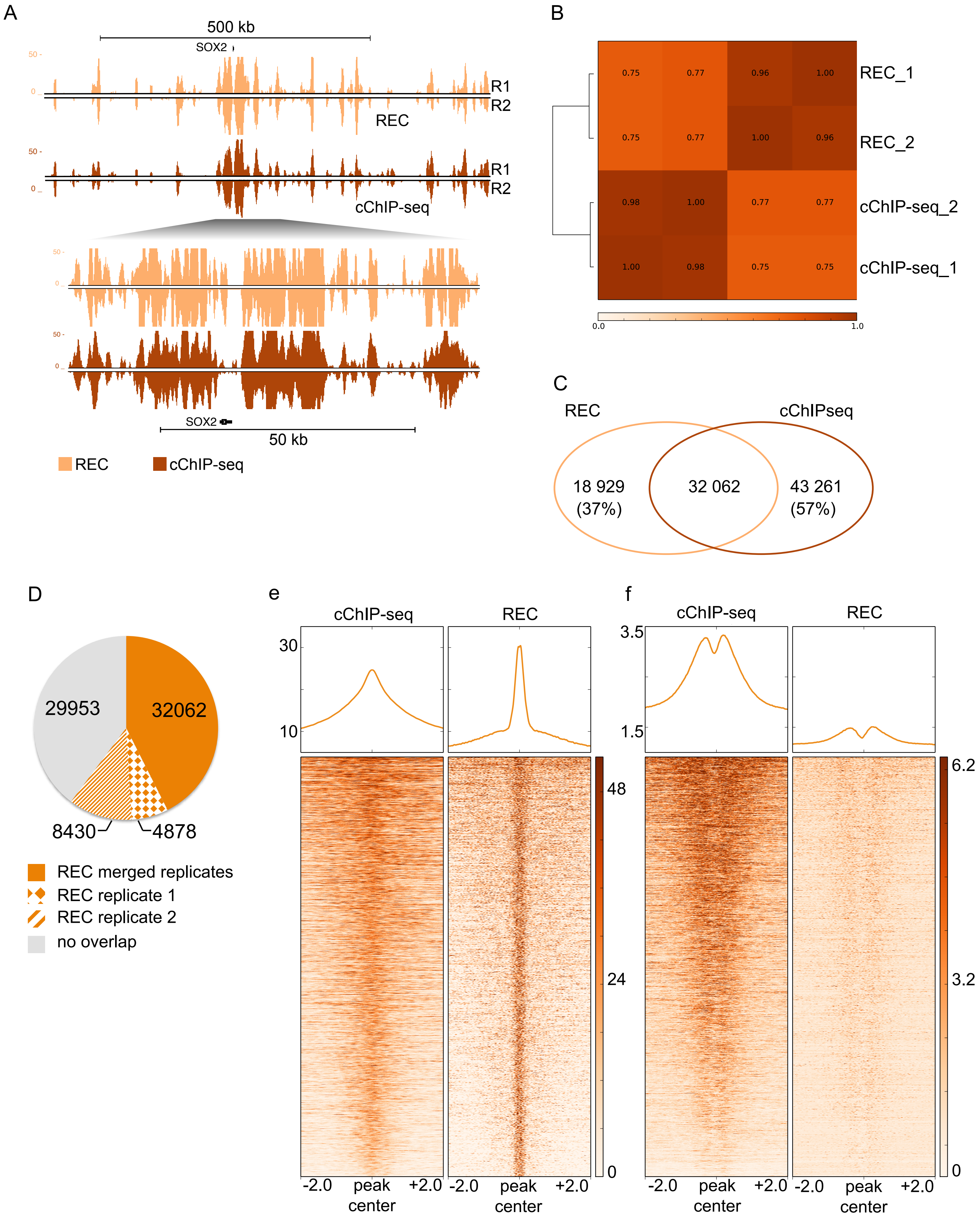
A
500 kb
SOX2
R1
R2
REC
R1
R2
cChIP-seq
SOX2
50 kb
REC
cChIP-seq
B
REC_1
REC_2
cChIP-seq_2
cChIP-seq_1
0.75 0.77 0.96 1.00
0.75 0.77 1.00 0.96
0.98 1.00 0.77 0.77
1.00 0.98 0.75 0.75
0.0
1.0
C
REC
cChIPseq
18 929 (37%)
32 062
43 261 (57%)
D
29953
32062
8430
4878
REC merged replicates
REC replicate 1
REC replicate 2
no overlap
e
cChIP-seq
REC
30
10
48
24
0
-2.0
peak center
+2.0
f
cChIP-seq
REC
3.5
1.5
6.2
3.2
0

Figure S5. cChIP-seq for H3K4me1 on 10,000 cells in H1 hESC line. A) UCSC genome browser snapshot showing H3K4me1 signal (RPKM input normalized) for cChIP-seq and REC data at the *SOX2* locus (chr3). R1: replicate 1; R2: replicate 2. B) Pearson's correlation values heatmap for replicates of cChIP-seq on 10,000 cells and REC data. C) Venn diagram shows the number of unique and common regions enriched for H3K4me1 across cChIP-seq and REC. In parenthesis is the percentage of unique regions per each dataset. D) Pie chart depicts the number of cChIP-seq peaks that overlap REC peaks called on merged replicated, REC peaks called on replicate 1 and REC peaks called on replicate 2. For peaks called on single replicate, only peaks that were not called on the merged replicate were counted. E) Heatmap representation of the signal intensity (RPKM input normalized) across cChIP-seq and REC data in a 4 kb window centered at REC peaks called on merged replicates (shown in C). F) Heatmap representation of the signal intensity (RPKM input normalized) across cChIP-seq and REC data in a 4 kb window centered at H1 enhancers predicted by RFECS (55,382 regions).
